# The human complement inhibitor Sushi Domain-Containing Protein 4 (SUSD4) expression in tumor cells and infiltrating T cells is associated with better prognosis of breast cancer patients

**DOI:** 10.1186/s12885-015-1734-7

**Published:** 2015-10-19

**Authors:** Emelie Englund, Bart Reitsma, Ben C. King, Astrid Escudero-Esparza, Sioned Owen, Akira Orimo, Marcin Okroj, Lola Anagnostaki, Wen G. Jiang, Karin Jirström, Anna M. Blom

**Affiliations:** 1 Department of Translational Medicine, Division of Medical Protein Chemistry, Lund University, Inga Maria Nilssons gata 53, 20502 Malmö, Sweden; 2 Cardiff’s China Medical Research Collaborative (CCMRC), Cardiff University School of Medicine, Cardiff University, Cardiff, UK; 3 Department of Pathology and Oncology, Juntendo University School of Medicine, Tokyo, Japan; 4 Department of Medical Biotechnology, Intercollegiate Faculty of Biotechnology UG‑MUG, Medical University of Gdańsk, 80210 Gdańsk, Poland; 5 Department of Clinical Pathology, Skåne University Hospital, Malmö, Sweden; 6 Department of Clinical Sciences Lund, Division of Oncology and Pathology, Lund University, Lund, Sweden

**Keywords:** Breast cancer, Cell migration, Complement inhibitor, Immunity

## Abstract

**Background:**

The human Sushi Domain-Containing Protein 4 (SUSD4) was recently shown to function as a novel inhibitor of the complement system, but its role in tumor progression is unknown.

**Methods:**

Using immunohistochemistry and quantitative PCR, we investigated SUSD4 expression in breast cancer tissue samples from two cohorts. The effect of SUSD4 expression on cell migration and invasion was studied *in vitro* using two human breast cancer cell lines overexpressing SUSD4.

**Results:**

Tissue stainings revealed that both tumor cells and tumor-infiltrating cells expressed SUSD4. The highest SUSD4 expression was detected in differentiated tumors with decreased rate of metastasis, and SUSD4 expression was associated with improved survival of the patients. Moreover, forced SUSD4 expression in human breast cancer cells attenuated their migratory and invasive traits in culture. SUSD4 expression also inhibited colony formation of human breast cancer cells cultured on carcinoma-associated fibroblasts. Furthermore, large numbers of SUSD4-expressing T cells in the tumor stroma associated with better overall survival of the breast cancer patients.

**Conclusion:**

Our findings indicate that SUSD4 expression in both breast cancer cells and T cells infiltrating the tumor-associated stroma is useful to predict better prognosis of breast cancer patients.

## Background

Sushi Domain-Containing Protein 4 (SUSD4), described so far in only two scientific papers, is a poorly studied human protein. The protein is predicted to be expressed as two different isoforms, where one is membrane-bound (SUSD4a) and the other soluble (SUSD4b). SUSD4a is a 49 kDa protein composed of four CCP (complement control protein) domains, a transmembrane region, and a cytoplasmic tail. SUSD4b is a smaller isoform (27 kDa) consisting of three CCP domains and a region of unknown homology. The protein may be further N-glycosylated at three predicted sites. Both isoforms are quite broadly expressed on mRNA level in many human tissues. We have previously demonstrated that SUSD4 functions as a complement inhibitor [1] but other possible functions of this protein remain unclear. In our previous study, we detected SUSD4 positive tumor-infiltrating cells in colon, lung and breast cancer, suggesting that SUSD4 might play a role in cancer progression.

It is unclear if complement and its regulators are beneficial or detrimental for the progression of cancer. As of yet, no clear consensus has been reached and the literature shows evidence of both hypotheses [2]. Complement can kill certain types of cancer cells. Because of this, cancer cells protect themselves against complement attack by expressing soluble or membrane-bound complement inhibitors [3, 4]. This is true for the widely expressed membrane-bound complement inhibitors, for example CD46 and CD59. On the other hand, complement activation can aid cancer progression by the production of C5a [5] and the creation of a chronic inflammatory environment. This means that complement can be beneficial or detrimental to cancer development, or perhaps both depending on the circumstances. Most likely, the outcome of complement activation on cells will highly depend on the environment and will differ for solid versus blood tumors. Therefore we now aimed to assess the expression of the complement inhibitor SUSD4 in human breast cancer and to determine if the degree of expression may be related to clinical prognosis.

Here, we show that SUSD4 is expressed by epithelial tumor cells, in which it affects migration and invasion, and in tumor-infiltrating CD8^+^ and CD4^+^ T cells. Furthermore, expression of SUSD4 is associated with an improved prognosis for breast cancer patients.

## Methods

### Immunohistochemical staining of breast cancer tissue microarrays (TMAs)

Tissue samples obtained from a cohort of 144 women diagnosed with breast cancer in Skåne, Sweden [6] were stained with rabbit anti-SUSD4 (home-made) as previously validated and described [1]. Ethical permission was obtained from the Lund University Regional Ethics Board, ref. no. 445/2007 whereby written consent was not required and patients were offered the option to opt out. The intensity of the SUSD4-specific signal in tumor cells was scored 0 (no expression), 1 (low expression) or 2 (high expression) independently by two scientists and one experienced clinical pathologist, who were all blinded with regard to clinical information. For statistical analyses, the scores were grouped into SUSD4 negative (score 0) and SUSD4 positive tumors (scores 1–2). In the stroma of the tumors, SUSD4 positive tumor-infiltrating cells were detected. The cells were counted for the whole tissue section and grouped into 0–15 (low) or 16–100 (high) SUSD4^+^ cells/section. Kaplan-Meier analyses and Breslow tests were used to determine the effect of SUSD4 expression by tumor cells or infiltrating cells on cancer-specific survival and recurrence-free survival. Uni- and multivariable Cox proportional hazard models based on SUSD4 expression were used to determine hazard ratios (HR) for cancer-specific death. Immunohistochemical data regarding hormone receptor status, Ki-67 and human epidermal growth factor receptor 2 (HER2) expression were available from previous studies [6, 7]. Definitions of estrogen receptor (ER) and progesterone receptor (PR) negativity followed current Swedish clinical guidelines (<10 % positive nuclei). Ki-67 status was assessed based on the percentage of positively stained nuclei and dichotomized into ≤25 % and >25 %. HER2 status was assessed by semiquantitative analysis according to a standard protocol [8]. Specimens were grouped as weakly (scores 0–2) and strongly expressing HER2 (score 3). Any differences in the distribution of clinical parameters and SUSD4 expression were calculated for tumor cells and tumor-infiltrating cells by using 2-tailed Mann–Whitney U tests. Exact p-values <0.05 were considered statistically significant. The calculations were performed using SPSS Statistics v. 22 (IBM). The original slides were scanned with an Aperio ScanScope slide scanner and representative pictures (40X magnification) were obtained in the ImageScope software (Aperio).

### SUSD4 RNA transcript analysis in a breast cancer cohort

Fresh frozen mammary tissues (normal, n = 32 and tumour, n = 127) from patients with breast cancer were collected immediately after surgery, under the research ethics approval from the Southeast Wales Research Ethics Committee and with informed consent, and stored at −80 °C until used. Patients were followed up routinely in the clinics with a median follow-up at 120 months, and their clinical characterisation was published previously [9]. The tissues were homogenized and total RNA was purified. cDNA was synthesized from 1 μg RNA according to the manufacturer’s specifications (AbGene). A qPCR was set up as described in [9], using the following primers for *SUSD4*: 5’-AAAACCTTATCTGGTCGTC-3’ and 5’-ACTGAACCTGACCGTACATCTCCGTGACTCACCATT-3’. A standard of *cytokeratin-19 (CK19)* was run simultaneously using primers 5’-CAGGTCCGAGGTTACTGAC-3’ and 5’-ACTGAACCTGACCGTACACACTTTCTGCCAGTGTGTCTTC-3’. The standard was used to obtain the transcript levels. The data were analyzed by Kaplan-Meier followed by Breslows test to determine if *SUSD4* transcript levels affected cancer-specific survival or recurrence free survival. *SUSD4* transcript levels were correlated to clinical parameters using Mann–Whitney U tests.

### Cells

Breast cancer cell lines MDA-MB-231 and BT20 (American Type Culture Collection, ATCC) were cultured in DMEM high glucose (Thermo Scientific) medium supplemented with 10 % fetal bovine serum (FBS), penicillin and streptomycin. Cells were frozen immediately after re-cultivation of the original aliquot, and all the experiments were performed on cultures originating from these secondary aliquots within no more than 5 passages. Cells were *Mycoplasma* negative and tested monthly for contamination with the VenorGEM Classic kit (Minerva Biolabs). Although SUSD4 is predicted to be expressed as two isoforms, we focused this study only on the cancer-related functions of the membrane-bound SUSD4a, which is the isoform easily detectable at protein level. Full-length SUSD4a [1] was cloned into the pcDNA3 vector (Life technologies) using restriction sites EcoRI and XhoI. The construct or empty vector (mock) were transfected to MDA-MB-231 and BT20 cells using lipofectamine 2000 (Life technologies) and clones were selected with G418 (Life technologies). Cell pellets were collected and RNA was purified with the RNeasy kit (Qiagen). cDNA was synthesized from 1 μg RNA by using 2.5 μM oligo(dT) primer, 24 U RnaseOUT, and 200 U Superscript III reverse transcriptase (Life technologies). A qPCR was set up using 10 ng cDNA/well in triplicate for each sample. Specific primers detecting *SUSD4a* (Hs01042141_m1), *cyclophilin A* (Hs99999904_m1), *TATA-box binding protein* (Hs00427621_m1), and *hypoxanthine phosphoribosyltransferase 1* (HPRT-1; Hs99999909_m1) were bought from Applied Biosystems. SUSD4a expression relative to the geometrical mean of the three references was calculated according to the ΔCt method [10].

SUSD4a protein expression was analysed by flow cytometry and western blot. For flow cytometry, 200 000 cells/well were incubated with 5 μg/ml anti-SUSD4 diluted in binding buffer (10 mM HEPES, 140 mM NaCl, 5 mM KCl, 1 mM MgCl_2_, 2 mM CaCl_2_, 0.02 % w/v NaN_3_, pH 7.2) for 1 hour at RT. The cells were washed in binding buffer, incubated with a secondary antibody conjugated to fluorescein isothiocyanate (FITC) for 30 min at RT, then resuspended in binding buffer and analysed by flow cytometry (Partec CyFlow Space flow cytometer) and the FlowJo software. For the western blot, lysates were run on a 12 % SDS-PAGE under reducing conditions. The gel was blotted (Trans-Blot Turbo, Bio-Rad) to a PVDF membrane, stained with 0.1 μg/ml anti-SUSD4 followed by a secondary antibody conjugated to horseradish peroxidase (HRP) and developed with ECL (Millipore).

### Growth assay

Cells (6000 cells/well) were plated out in duplicates in four identical 96-well plates (Nunc). The plates were incubated for 0.5 h, 24 h, 72 h, or 96 h, before cell fixation with 4 % formaldehyde and staining with 0.5 % w/v crystal violet. Excess dye was washed away with tap water and the plate was left to dry over night. The dye was extracted with 10 % acetic acid and the absorbance was read at 540 nm using a microplate reader (Cary50Bio, Varian). The data were normalized to the highest value of each repetition.

### Adhesion

A layer of matrigel (5 μg/well, BD Biosciences) was coated in quadruplicates in a 96-well plate. After drying and rehydration of the matrigel, cells (MDA-MB-231; 3x10^4^ cells/well and BT20; 5x10^4^ cells/well) were allowed to bind for 45 min at 37 °C. Unbound cells were removed by washing with BSS (680 mM NaCl, 15 mM KCl, 7 mM KH_2_PO_4_, 3.5 mM Na_2_HPO_4_, pH 7.2). The cells were fixed with 4 % formaldehyde and stained with 0.5 % w/v crystal violet as described above. After the plate was dry, two random pictures were taken of each well (40X objective; EVOS FL inverted microscope) and the cells were counted (ImageJ).

### Wound healing assay

Cells were grown to confluency in a 6-well plate (Nunc). Two scratches were made per well with a sharpened yellow pipette tip. Pictures were taken at three positions (exactly the same position every time using a 10X objective) along each scratch at 0 h, 3 h and 6 h. The wound area was measured in ImageJ and the average of the 6 positions was used. The data were normalized to the wound area at 0 h for each sample.

### Migration and invasion

Cells were resuspended in DMEM high glucose medium supplemented with 1 % FBS and placed in plain inserts (migration, 8 microns, BD Biosciences) in duplicates or inserts coated with matrigel (invasion, 8 microns, BioCoat, Corning) in singlet. The inserts were placed (before the addition of cells) in wells containing DMEM high glucose supplemented with 10 % FBS. Cells (MDA-MB-231; 5x10^4^ cells/well and BT20; 10x10^4^ cells/well) were left to migrate/invade for 22 h (MDA-MB-231) or 44 h (BT20). Cells that had moved to the underside of the inserts were fixed with formaldehyde and stained with crystal violet. Four pictures were taken of each inserts using a 40X objective and the number of migrating/invading cells was quantified with ImageJ.

### Clonogenic co-culture assay

Carcinoma-associated fibroblasts (CAFs) or control originating from breast tissue [11] were cultured O/N in triplicates in a 96-well plate (5x10^3^ cells/well). SUSD4a- or mock-transfected BT20 cells (100 cells/well) were added to each well and were cultured for 10 days. The cells were fixed with 70 % ethanol and stained with 0.1 % w/v toluidine blue (Sigma-Aldrich). Excess dye was removed with BSS and the plate was left to dry O/N. A picture of each well was taken using a 4X objective and the average cluster size of the colonies was quantified with ImageJ. Additional representative pictures were taken using a 40X objective.

### Adhesion of cancer cells to CAFs

CAFs were grown to confluency in quadruplicates in a 96-well plate before the addition of SUSD4a- or mock-transfected MDA-MB-231 or BT20 cells (30x10^3^ cells/well). The plate was incubated for 45 min and unbound cells were removed with BSS. Adherent cells were fixed with formaldehyde and stained with toluidine blue. The plate was rinsed with tap water and allowed to dry O/N at RT. Two random pictures were taken of each well using a 40X objective and the number of adherent cancer cells was counted using ImageJ.

### Fluorescence microscopy

Breast cancer TMAs (as described above) were double-stained for SUSD4 (2 μg/ml) and CD3 (10 μg/ml; clone OKT3; eBioscience), CD4 (8 μg/ml; clone 4B12; Dako) or CD8 (3 μg/ml; clone C8/144B; Dako) as described previously [12]. Pictures were taken with a Zeiss LCM 510 confocal microscope (20X objective).

### Purification of T cells

Peripheral blood was drawn from healthy volunteers according to the permit of the local ethical committee in Lund, Sweden. Peripheral blood mononuclear cells (PBMCs) were isolated by centrifugation over a density gradient (Lymphoprep, Stemcell technologies). CD4^+^ or CD8^+^ T cells were purified from the PBMCs by using specific kits (Miltenyi Biotec). Wells (48-well plate) were coated O/N at 4 °C with a mix of 2 μg/ml anti-CD3 and 2 μg/ml anti-CD28 (clone CD28.2; eBioscience) diluted in PBS. The isolated T cells were either immediately frozen at −80 °C (fresh) or stimulated for 24 h in coated wells in RPMI medium (Thermo Scientific) supplemented with 10 % FBS and 5 U/ml IL-2 (Immunotools). Fresh and stimulated T cells were used for SUSD4a expression analysis simultaneously by both qPCR and western blot. The experiment was performed three times using blood from different donors.

RNA was purified (RNeasy, Qiagen) and cDNA was synthesized as described above. *SUSD4a* gene expression was analysed by qPCR using 10 ng cDNA/well as described above. Three different primers specific for *SUSD4a* (Hs01042141_m1, Hs01047294_m1, Hs01047293_m1), and the reference primers (Applied Biosystems) *cyclophilin A*, *beta-2 microglobulin* (*B2M*, Hs00984230_m1), and *HPRT-1* were used.

The fresh or stimulated T cells were lysed in RIPA buffer and 10 μg total protein/sample was analysed by 12 % SDS-PAGE under reducing conditions. The gel was blotted and the membrane was stained as described above. After development of the SUSD4a signal, the lower part of the blot was washed and stained for the loading control B2M (17 ng/ml; Abcam, ab75853).

## Results

### SUSD4 expression is associated with better survival at both protein and RNA levels

**Fig. 1:**
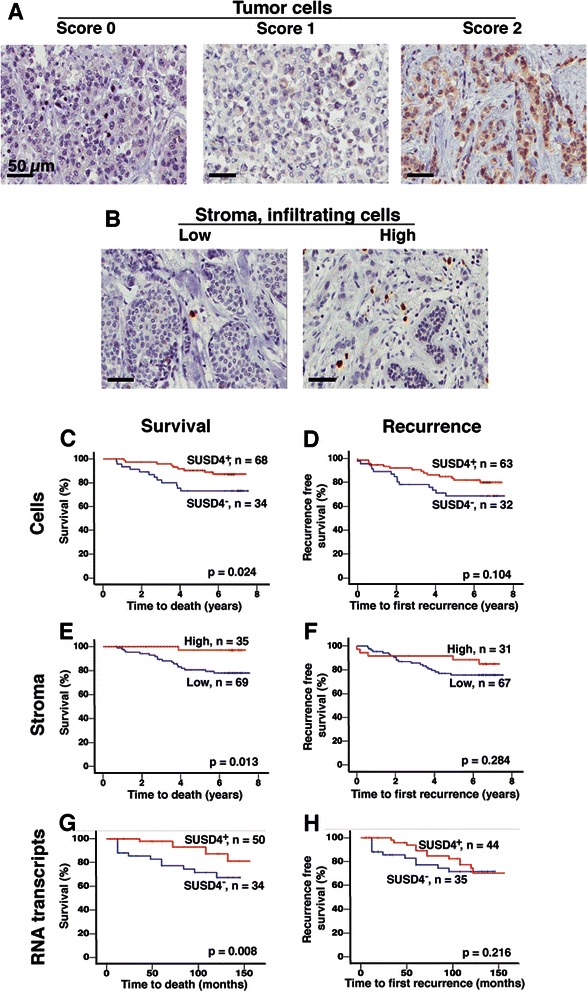
SUSD4 expression in breast cancer is associated with an improved prognosis. A cohort of breast cancer tissue samples were stained for SUSD4, and the intensity of the staining in tumor cells was scored 0, 1, or 2. Representative pictures were taken at 40X magnification (**a**). The scores were grouped into SUSD4^+^ (1, 2) or SUSD4^−^ (0) tumors. The number of SUSD4^+^ infiltrating cells was counted in each section, and was grouped into low (0–15 cells/section) and high (16–100 cells/section). Representative pictures are shown in **b**. Kaplan-Meier analyses showed that SUSD4 expression by tumor cells was associated with prolonged breast cancer specific survival of the patients (**c**), but recurrence free survival was not affected (**d**). A higher number of SUSD4^+^ infiltrating cells in the stroma was also beneficial for the survival rate (**e**), while it did not affect recurrence free survival (**f**). SUSD4 transcript levels were analysed by qPCR in a second cohort of breast cancer samples. Increased levels of SUSD4 transcripts was associated with improved survival (**g**), but not with recurrence free survival (**h**).

The intensity of the SUSD4 staining in tumor cells was scored 0, 1 or 2 (Fig. 1a) and the number of SUSD4^+^ infiltrating cells in each section was counted (Fig. 1b). Kaplan-Meier analyses and Breslow tests showed that SUSD4 expression in tumor cells and in the tumor infiltrating cells had a positive effect on the breast cancer specific survival rate of the patients (Fig. 1c, e), but did not significantly affect recurrence free survival (Fig. 1d, f).

Associations between SUSD4 expression in tumor cells and clinical parameters showed that SUSD4^+^ tumors had a tendency (p = 0.066) to be smaller (Table 1). The SUSD4^+^ tumors were more differentiated (NHG; p < 0.001), and less prone to lymph node metastasis (nodal status; p = 0.027) as compared to SUSD4^−^ tumors.

**Table 1:** Associations between SUSD4 and clinical parameters

Factor		Patient	SUSD4^+^ tumor cells			Patient	SUSD4^+^ infiltrating cells		
			No	Yes	*P* ^*^		0-15	16-100	*P* ^*^
		*N*				*N*			
All		123	46 (37)	77 (63)		123	87 (71)	36 (29)	
Age at diagnosis		123			0.047	123			0.006
Median (min, max)			63 (34, 97)	67 (35, 91)			68 (34, 97)	61 (41, 79)	
Tumor size,		123			0.066	123			
Median mm (min, max)			23.5 (10, 73)	21 (7, 145)			23 (8, 145)	19.5 (8, 73)	0.143
NHG					<0.001				0.045
I	*N (%)*	15	2 (13)	13 (87)		17	11 (65)	6 (35)	
II		58	16 (28)	42 (72)		56	35 (63)	21 (37)	
III		50	28 (56)	22 (44)		50	41 (82)	9 (18)	
Nodal status					0.027				0.853
0	*N (%)*	58	16 (28)	42 (72)		60	42 (70)	18 (30)	
1-3		34	14 (41)	20 (59)		32	23 (72)	9 (28)	
>4		18	10 (56)	8 (44)		18	13 (72)	5 (28)	
Missing		13				13			
ER status					0.008				0.041
Negative	*N (%)*	18	12 (67)	6 (33)		17	16 (94)	1 (6)	
Positive		105	34 (32)	71 (68)		106	71 (67)	35 (33)	
PR status					0.117				0.295
Negative *N (%)*		40	19 (48)	21 (52)		40	31 (78)	9 (22)	
Positive		83	27 (32)	56 (68)		83	56 (68)	27 (32)	
HER2					0.013				1.000
Negative	*N (%)*	112	38 (34)	74 (66)		106	77 (73)	29 (27)	
Positive		9	7 (78)	2 (22)		11	8 (73)	3 (27)	
Missing		2				6			
Ki67					0.239				0.136
0-24 %	*N (%)*	50	17 (34)	33 (66)		52	34 (65)	18 (35)	
>25 %		58	27 (47)	31 (53)		57	45 (79)	12 (21)	
Missing		15				14			

^*^Mann–Whitney, 2-tailed Exact p-value

Cox uni- and multivariable analyses were performed in order to determine the prognostic value of SUSD4 expression in tumor cells (Table 2). The results indicated that SUSD4 was an independent biomarker for cancer-specific survival (p = 0.040; HR: 0.34; 95 % CI: 0.1 – 0.9).

**Table 2:** Cox uni- and multivariate analyses of associations between SUSD4 expression and known predictive markers for survival.

Survival tumor cells	Univariate				Multivariate			
Variable	*N*	HR	95 % CI	*P*	*N*	HR	95 % CI	*P*
SUSD4 (no vs. yes)	122	0.41	0.2-0.9	0.042	107	0.34	0.1-0.9	0.040
Age at diagnosis	144	1.05	1.0-1.1	0.003	107	1.05	1.0-1.1	0.008
ER (neg vs. pos)	144	0.23	0.1-0.5	0.001	107	0.40	0.1-1.1	0.090
NHG	144	5.14	2.0-13.0	0.001	107	1.60	0.4-5.8	0.478
Size (<20 vs. >20 mm)	144	3.04	1.2-7.8	0.020	107	2.37	0.6-9.1	0.209
Ki67 (weak vs. strong)	124	4.26	1.4-12.8	0.010	107	1.65	0.4-6.6	0.483
Survival infil. cells	Univariate				Multivariate			
Variable	*N*	HR	95 % CI	*P*	*N*	HR	95 % CI	*P*
SUSD4 (low vs. high)	122	0.12	0.02-0.9	0.039	108	0.25	0.03-2.0	0.192
Age at diagnosis	144	1.05	1.0-1.1	0.003	108	1.03	1.0-1.06	0.125
ER (neg vs. pos)	144	0.23	0.1-0.5	0.001	108	0.56	0.2-1.7	0.318
NHG	144	5.14	2.0-13.0	0.001	108	2.14	0.6-7.8	0.250
Size (<20 vs. >20 mm)	144	3.04	1.2-7.8	0.020	108	2.18	0.6-8.6	0.265
Ki67 (weak vs. strong)	124	4.26	1.4-12.8	0.010	108	1.20	0.3-5.6	0.815

A higher number of SUSD4^+^ infiltrating cells in the tumor stroma were associated with a diagnosis at an earlier age (p = 0.006). It was also associated with more differentiated (NHG; p = 0.045) and ER positive (p = 0.041) tumors (Table 1). The SUSD4 expression in infiltrating cells was significantly associated with survival in the univariable Cox analysis (p = 0.039; HR: 0.12; 95 % CI: 0.02 – 0.9), but not in the multivariable test (p = 0.192; HR: 0.25; 95 % CI: 0.03 – 2.0, Table 2) indicating that it was not an independent predictor of survival.

RNA was purified from breast cancer tissues isolated from patients included in a second independent cohort, and *SUSD4* transcript levels were analysed by qPCR. Kaplan-Meier analyses showed that the presence of *SUSD4* transcripts in the tumor tissues was significantly associated with improved cancer specific survival (p = 0.009; Fig. 1g), but not recurrence free survival (p = 0.220; Fig. 1h). The Nottingham Prognostic Index (NPI) tended to be lower for tumors with higher levels of SUSD4 transcripts (p = 0.054), which indicated that 85 % of patients with SUSD4-expressing tumors survived for at least 5 years (NPI score ≤3.4; compared to score ≥5.4 which represented a 5 year survival rate of 50 %). Tumors expressing high levels of *SUSD4* transcripts tended to be less invasive (p = 0.056; negative vs. positive nodal status).

### SUSD4a affects the long-term growth of cancer cells

**Fig. 2:**
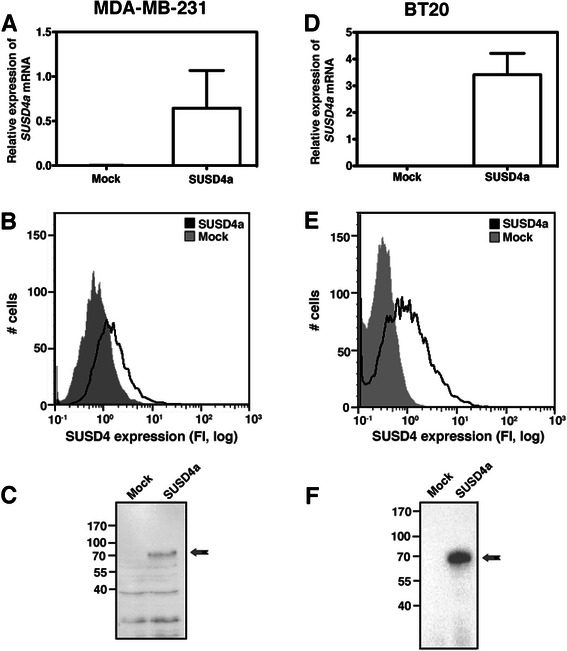
Expression of SUSD4 in two breast cancer cell lines. SUSD4a was overexpressed in the MDA-MB-231 and BT20 cell lines. The expression was verified at RNA level by qPCR (**a**, **d**), and at protein level by flow cytometry (**b**, **e**) and western blot of cell lysates (**c**, **f**). The data in the Fig. represents three independent repetitions ± SD (**a**, **d**), representative histograms (**b**, **e**) and blots (**c**, **f**)

The breast cancer cell lines MDA-MB-231 and BT20 were stably transfected with SUSD4a cDNA or empty vector (mock). The RNA expression was verified by qPCR using a specific primer (Fig. 2a, d). Protein expression was tested by flow cytometry (Fig. 2b, e) and by western blot (Fig. 2c, f). All three experiments verified the expression of SUSD4a in both cell lines.

**Fig. 3:**
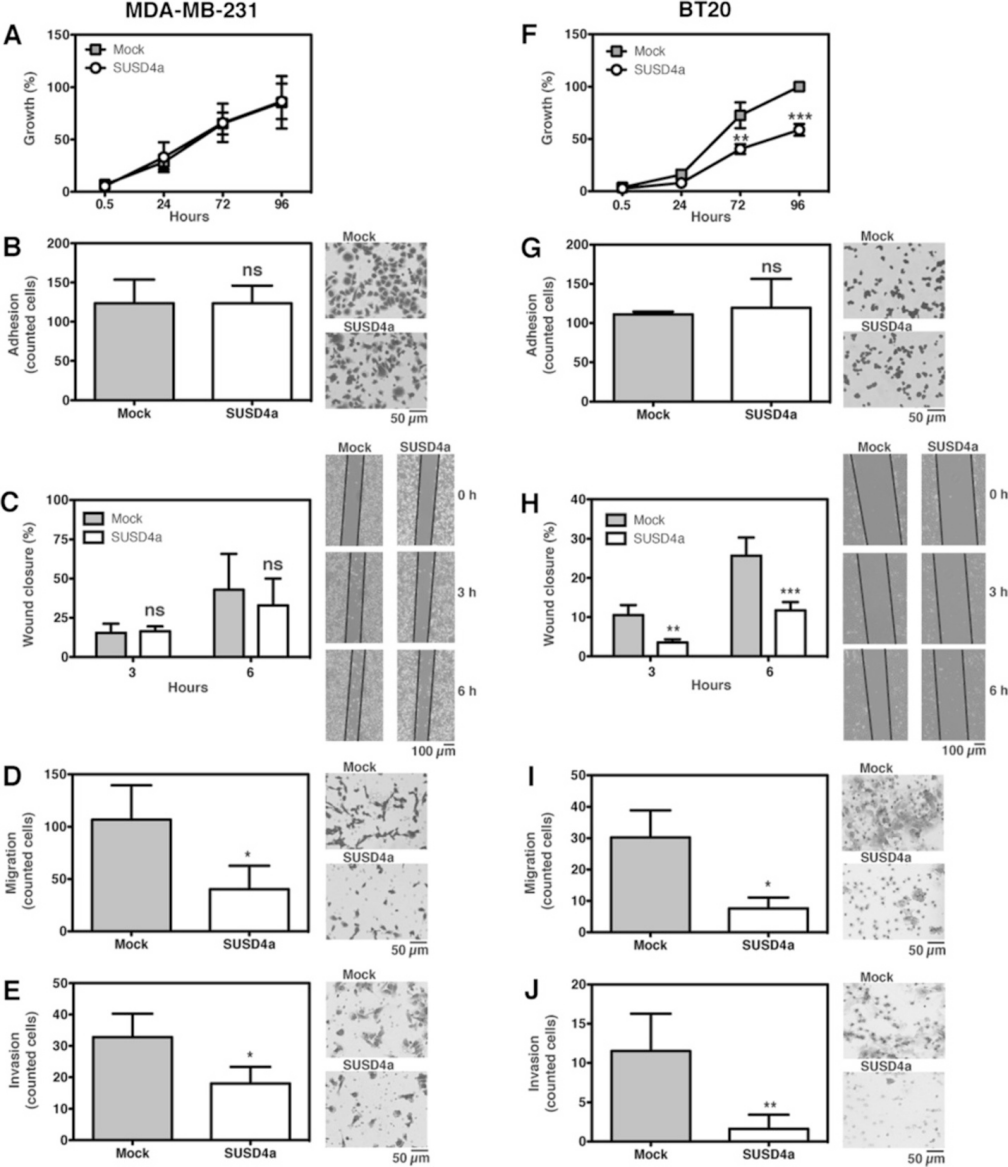
SUSD4 is involved in cell migration and invasion. To determine the role of SUSD4 in breast cancer, several functional assays were preformed. The growth assay showed no difference between SUSD4a- and mock- transfected MDA-MB-231 (**a**), while the SUSD4a-transfected BT20 cells grew slower after 72 hours (**f**). The ability of cancer cells to adhere to matrigel was not affected by SUSD4a (**b**, **g**), but the protein could influence migration. Random migration was decreased in BT20 cells expressing SUSD4a after 3 hours (**h**), but not in MDA-MB-231 cells (**c**). SUSD4a decreased both directed migration (**d**, **i**), along a gradient of serum, and invasion through a layer of matrigel (**e**, **j**) in both tested cell lines. The data represents at least three independent experiments ± SD. SUSD4a was compared to mock by 2-way ANOVA with Bonferroni post-test (**a**, **c**, **f**, **h**), or unpaired Student’s t-test (**b**, **d**, **e**, **g**, **i**, **j**). The symbols ns, *, **, and *** stand for not significant, p < 0.05, p < 0.01, and p < 0.001

No statistically significant differences were observed when comparing the growth rate of SUSD4a- or mock-transfected MDA-MB-231 cells (Fig. 3a). For BT20 cells, there were no differences after 24 hours but after 72 and 96 hours a significantly decreased growth rate was observed for cells expressing SUSD4a (Fig. 3f).

### SUSD4a is not involved in adhesion

To determine if SUSD4 alters the adhesive properties of the cancer cells, adhesion to matrigel was tested. The results showed no significant differences between the SUSD4- and mock-transfected cells (Fig. 3b, g).

### Expression of SUSD4a slows migration and invasion of cancer cells

Both the wound healing assay and the migration assay were used to test if SUSD4 influences cell motility. In the wound healing assay, cell movement was random and a significant difference between SUSD4a- and mock-transfected BT20 cells was observed after 3 hours (Fig. 3h), but not for the MDA-MB-231 cell line (Fig. 3c). In the migration assay, where the cells migrated towards higher serum concentrations, both MDA-MB-231 and BT20 cells transfected with SUSD4a moved significantly slower as compared to the mock (Fig. 3d, i).

The invasion assay tests both migration along a serum gradient and the ability of the cells to digest through a layer of extracellular matrix (Matrigel). A decreased ability to invade was observed for both cell lines expressing SUSD4 as compared to mock-transfected cells (Fig. 3e, j).

### Cancer cells expressing SUSD4a form smaller colonies when co-cultured with CAFs

**Fig. 4:**
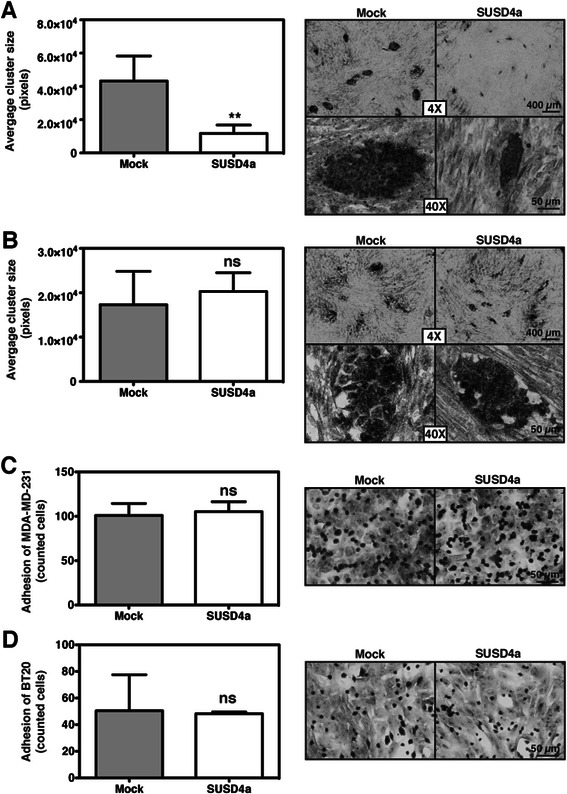
SUSD4a expressing BT20 cells form smaller colonies. The clonogenic potential of BT20 cells expressing SUSD4a was tested in co-culture with fibroblasts. SUSD4a-transfected BT20 cells formed significantly smaller colonies in co-culture with CAFs (**a**) but not with control fibroblasts (**b**). The ability of the SUSD4a-transfected cells to adhere to CAFs was also tested. No significant differences were observed for either MDA-MB-231 (**c**) or BT20 (**d**). The graphs represent data collected from at least three independent experiments ± SD; ns and ** stand for not significant and p < 0.01, respectively

The ability of BT20 cells to form colonies on a monolayer of fibroblasts was assessed in a clonogenic assay. BT20 cells expressing SUSD4a formed significantly smaller colonies when in co-culture with CAFs (Fig. 4a), but not with control fibroblasts (Fig. 4b). The MDA-MB-231 cell line did not form colonies when co-cultured with CAFs or control fibroblasts. The smaller size of the colonies was not due to decreased adhesion of the SUSD4a expressing cancer cells to CAFs, as was shown for both MDA-MB-231 (Fig. 4c) and BT20 cells (Fig. 4d).

### SUSD4 is expressed by tumor-infiltrating T cells

**Fig. 5:**
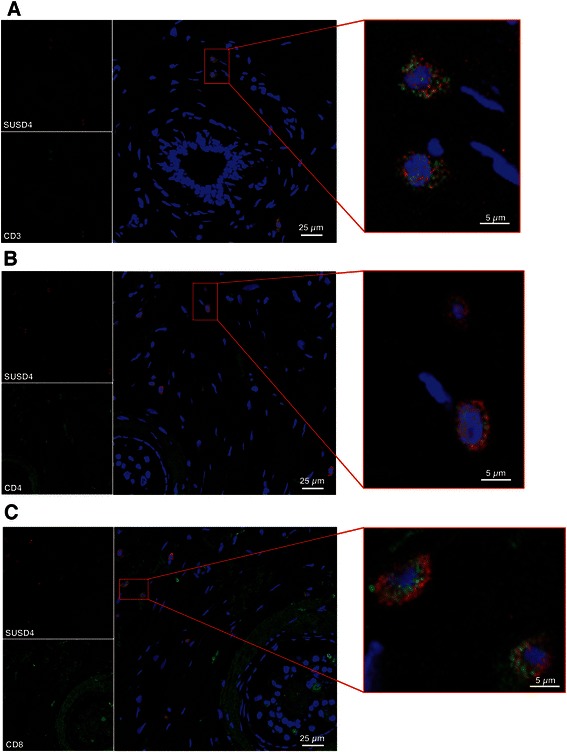
SUSD4 is expressed by tumor-infiltrating T cells. In order to determine what type of infiltrating cells express SUSD4, fluorescent double stainings were preformed. Staining with anti-SUSD4 and anti-CD3 showed that SUSD4^+^ infiltrating cells are T cells (**a**). Both CD4^+^ (**b**) and CD8^+^ (**c**) T cells are positive for SUSD4

In order to determine which type of stromal infiltrating cells expressed SUSD4, fluorescent double-stainings were performed on the human breast cancer sections. Stainings with anti-SUSD4 and anti-CD3 (Fig. 5a) revealed that these cells are T cells. Further stainings with anti-CD4 (Fig. 5b) or anti-CD8 (Fig. 5c), showed that both CD4^+^ and CD8^+^ T cells expressed SUSD4.

### SUSD4 expression by CD4^+^ and CD8^+^ T cells isolated from peripheral blood

**Fig. 6:**
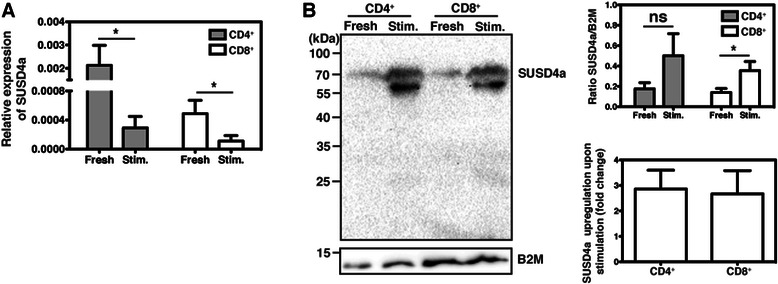
SUSD4a expression by T cells isolated from peripheral blood. The expression of SUSD4a by CD4^+^ and CD8^+^ T cells isolated from peripheral blood was tested by qPCR (**a**). Freshly purified (fresh) T cells expressed SUSD4a, but this expression was downregulated when the cells were stimulated (stim.). T cells isolated from the same donors, at the same time points, were also analysed for SUSD4a protein expression by western blot (**b**). Interestingly, SUSD4a protein was more than 2.5 fold upregulated in stimulated T cells. The data represents three donors ± SD and was analysed by Student’s T-test comparing fresh to stimulated. The symbols ns, and * stand for not significant, and p < 0.05

SUSD4 expression in T cells purified from peripheral blood was evaluated by qPCR and western blot. The qPCR and the western blot were performed on T cells isolated at the same time and each repetition represents one donor. *SUSD4a* gene expression was very similar for all three tested primers, and the results showed that *SUSD4a* mRNA expression was downregulated upon T cell stimulation (Fig. 6a, one representative primer is shown). However, SUSD4a protein was more than 2.5-fold upregulated in the stimulated T cells (Fig. 6b). These data suggest changes in regulation of SUSD4a expression during T cell activation and therefore a potential involvement of SUSD4a in T cell function.

## Discussion

SUSD4 is not a well-studied protein and there are only two published papers [1, 13] pertaining to the function of the protein. Here, we show that breast cancer epithelial cells express SUSD4 and that positive expression was significantly associated with a prolonged patient survival. This may be explained, at least in part, by our observation that SUSD4a affects cell migration, invasion and clonogenic ability. Furthermore, expression of SUSD4 on infiltrating T cells was identified and as expected, increased numbers of such cells was correlated with increased patient survival.

We have previously shown that both isoforms of SUSD4, a and b, produced as tagged recombinant proteins, inhibit complement by affecting the C3 convertase [1]. In addition, we detected SUSD4 expression on tumor infiltrating cells in several types of cancer. In this study, we aimed to identify the role of SUSD4 in breast cancer. For the *in vitro* assays, we transfected either SUSD4a or SUSD4b cDNA into the MDA-MB-231 and BT20 cell lines, and both cell lines showed high expression levels of SUSD4a or SUSD4b mRNA. When performing functional assays on these cells, both SUSD4 isoforms had very similar effect. However, we have not been able to detect the SUSD4b protein either in cell lysate or in conditioned medium. The specificity of our affinity purified polyclonal anti-SUSD4 antibody had already been validated [1]. The antibody was raised against the CCP domains common for both SUSD4 isoforms, and therefore it was not possible to distinguish between the isoforms in the tissue stainings. However, western blotting allowed us to determine the particular SUSD4 isoform based on the different molecular weight of the proteins. It is possible that the impaired detection of wild type SUSD4b is caused by a different folding of the protein devoid of the tag, or there is an additional regulation downstream of transcription. Nonetheless, since we could not detect expression of untagged SUSD4b protein, we opted not to show *in vitro* results pertaining to SUSD4b and only discuss the effects of SUSD4a.

*In vitro* functional studies revealed that SUSD4 expression decreased the growth rate of BT20 cells after 72 hours, but the growth rate of MDA-MB-231 was not affected. SUSD4 expression also decreased the migration and invasion of both cell lines. The effect of SUSD4 expression was more pronounced for BT20 cells. It is important to note that SUSD4 did not affect the cell cycle of the tested cell lines (data not shown). Taken together, these data suggest that SUSD4 independently inhibits both growth and migration of cancer cells. The decreased invasiveness of the cells was likely due to decreased migration. Co-cultures of BT20 cells with CAFs, but not with control fibroblasts, showed formation of smaller colonies of cells expressing SUSD4 as compared to mock. This assay primarily measured cell proliferation of the cancer cells, but since we only observed an effect of SUSD4 in co-culture with CAFs and not control fibroblasts, this cannot be the only important factor. Possibly, the CAFs secrete factors affecting the cancer cells and thereby enhance the anti-tumor effects of SUSD4.

In this study, we showed that SUSD4 expression in tumor cells and tumor-infiltrating cells was associated with higher survival rates of breast cancer patients. The SUSD4-expressing tumor-infiltrating cells were identified as CD4^+^ and CD8^+^ T cells. Although it is known that the presence of infiltrating T cells is beneficial for breast cancer-specific survival [14, 15], it has not been shown that these cells express SUSD4, which is here identified as a protein suppressing the aggressive phenotype of breast cancer cells. *SUSD4* mRNA expression in total peripheral blood CD4^+^ and CD8^+^ T cells was downregulated during stimulation, while SUSD4a protein expression was upregulated. This suggests a negative regulation of *SUSD4a* mRNA during T cell activation, which is not necessarily contradictory to increases in protein levels, if combined with decreases in post-transcriptional regulation of expression or protein turnover. The increased level of SUSD4a protein in activated T cells provides further explanations for the finding that levels of SUSD4^+^ infiltrating cells are associated with increased survival, as an active anti-tumor T-cell response would be beneficial to patients. It is of note that another complement receptor, CD46, which also consists of 4 CCP domains, functions as a strong co-stimulatory receptor on human T cells [16]. The function and factors controlling expression of SUSD4 on T cells therefore demand further investigation.

The knowledge about the role of complement in human diseases, including cancer, is still expanding. For a long time complement was believed to support the host against tumor cells, but new studies also show a pro-tumor activity of certain complement components [17]. Based on our present study it is hard to determine whether the tumor-suppressing effect of SUSD4 stems from the complement function of this protein or whether it is another, independent function. SUSD4 is not the first complement inhibitor implied to regulate tumor growth. The putative tumor suppressor CSMD1 was already shown to diminish complement activation at the level of C3b [18]. On the other hand, the expression of complement inhibitors by tumor cells has also been associated with enhanced tumor progression manifested by larger tumor size, lower differentiation rate, shorter survival time and shorter remission [19, 20].

Interestingly, another CCP domain-containing protein has also been identified to suppress the phenotype of tumors. SUSD2 expression by HeLa cells was shown to inhibit cell migration and invasion [21], although the SUSD2 and SUSD4 genes are located to different chromosomes (22q11 and 1q41, respectively). Additionally, SUSD2 contains only one CCP domains and is thus unlikely to inhibit complement, since such activity usually requires at least three consecutive CCP domains.

## Conclusion

In summary, SUSD4 expression by tumor cells improved the survival rate of breast cancer patients and led to decreased growth rate and migration of transfected cells *in vitro*. Furthermore, SUSD4 is expressed by CD4^+^ and CD8^+^ T cells in tumor stroma, which correlates with good prognosis. Although SUSD4 has previously been described as a novel complement inhibitor, it is likely that the protein’s anti-tumor effects are independent of its complement-related functions.
